# The Clinical Characteristics of Pheochromocytomas and Paragangliomas with Negative Catecholamines

**DOI:** 10.3390/jcm11195583

**Published:** 2022-09-23

**Authors:** Lin Zhao, Xiaoran Zhang, Xu Meng, Ting Zhang, Hua Fan, Qiongyu Zhang, Yecheng Liu, Xianliang Zhou, Huadong Zhu

**Affiliations:** 1 Department of Cardiology, Fuwai Hospital, National Center for Cardiovascular Disease, Chinese Academy of Medical Sciences and Peking Union Medical College, Beijing 100037, China; 2 Department of Emergency Medicine, Beijing Friendship Hospital, Capital Medical University, Beijing 100050, China; 3 Emergency Department, State Key Laboratory of Complex Severe and Rare Diseases, Peking Union Medical College Hospital, Chinese Academy of Medical Science and Peking Union Medical College, Beijing 100730, China; 4 Department of Family Medicine & Division of General Internal Medicine, Department of Medicine, State Key Laboratory of Complex Severe and Rare Diseases, Peking Union Medical College Hospital, Chinese Academy of Medical Sciences, Beijing 100730, China; 5 Department of Urology, State Key Laboratory of Complex Severe and Rare Diseases, Peking Union Medical College Hospital, Chinese Academy of Medical Science and Peking Union Medical College, Beijing 100730, China

**Keywords:** pheochromocytomas, paragangliomas, catecholamine

## Abstract

Pheochromocytomas and paragangliomas (PPGLs) associated with negative catecholamines are not uncommon. However, few studies have examined clinical features of patients with these tumors. In the absence of available data, it is difficult to identify characteristics of patients with potential PPGLs and normal serum and urine screens. Therefore, an analysis of patients with PPGLs was conducted retrospectively to compare the clinical features of patients with positive and negative catecholamines. This study included 214 patients, including 69 patients with negative catecholamines. Prevalence rates of diabetes (*p* < 0.001) and hypertension (*p* < 0.001) were lower and tumor diameter (*p* < 0.001) was smaller in the negative-catecholamine group compared with the positive-catecholamine group. Multivariable logistic regression analysis showed that extra-adrenal PPGLs were independently positively associated with negative catecholamines (*p* = 0.004); hypertension (*p* = 0.001) and tumor diameter (*p* = 0.016) were independently negatively associated with negative catecholamines. There was no significant difference in tumor recurrence between the two groups (mean follow-up, 20.54 ± 11.83 months) (*p* = 0.44). The results demonstrated that PPGL patients with negative catecholamines were more likely to have extra-adrenal tumors and less likely to have comorbidities, and these patients should also be closely monitored for tumor recurrence.

## 1. Introduction

Pheochromocytomas and paragangliomas (PPGLs) are rare neuroendocrine tumors. Pheochromocytomas (PHEOs) originate from chromaffin cells of the adrenal cortex, and paragangliomas (PGLs) originate from extra-adrenal chromaffin cells of the sympathetic paravertebral ganglia located in the thorax, abdomen, pelvis, and from parasympathetic ganglia located along the glossopharyngeal and vagal nerves in the neck and at the base of the skull [1]. The combined incidence is approximately 0.57 cases per 100,000 person-years [2]. Symptoms such as headache, palpitations, and sweating are caused by catecholamines produced by these tumors [3]. PPGLs should be diagnosed and treated as soon as possible because incorrectly treated PPGLs can cause life-threatening complications [4].

As PPGLs secrete catecholamines, the diagnostic biochemical tests for these tumors involve the detection of these hormones. According to current clinical practice guidelines, measurements of plasma or urinary catecholamines should be performed during biochemical screening for PPGLs, and there is no recommendation regarding which test should be preferred [1]. Even though these tests are with high sensitivity, for example, the sensitivity of plasma free metanephrines to diagnose PPGLs has been reported as between 96 and 99% [5], there are indeed many patients with PPGLs who do not exhibit elevated catecholamines. It may pose a problem for clinicians who mistakenly believe they have ruled out PPGLs. However, there is little information in the current literature concerning the clinical features of catecholamine-negative PPGLs, with the majority being case studies of single patients [6,7,8]. Therefore, the objective of this study was to compare clinical characteristics of PPGL patients with positive and negative catecholamine levels to provide more information for clinicians to better understand this clinical population.

## 2. Materials and Methods

All consecutive adult patients with PPGLs who underwent surgical resection and had their diagnosis confirmed by pathological examinations from January 2018 to June 2020 in the Peking Union Medical College Hospital were retrospectively enrolled. The electronic medical files of patients were reviewed. Clinical history data, preoperative biochemical examination results, and tumor diameters and locations were obtained from the electronic medical record. A total of 313 patients were eligible for study inclusion; we then excluded 58 patients with no catecholamine information, 25 patients who presented to our hospital due to recurrence or metastasis of PPGLs after treatment in other hospitals, and 16 patients with incomplete clinical data. In total, 214 were included for analysis.

Patients were grouped according to catecholamine concentration measurements. Negative catecholamine was defined as when 24 h urinary catecholamine (epinephrine and norepinephrine), plasma metanephrine, and plasma normetanephrine concentrations did not exceed their respective reference limits. Positive catecholamine was defined as an abnormal elevation of the 24 h urinary catecholamine (epinephrine and norepinephrine), plasma metanephrine, or plasma normetanephrine. The diagnosis of hypertension and diabetes was made on based on patient history and preoperative blood pressure and blood glucose measurements, respectively. The patterns of hypertension in patients with PPGLs comprised sustained, paroxysmal, and mixed patterns [3]. Recurrence was defined as local relapse detected on computed tomography, magnetic resonance imaging, or functional imaging. Metastatic PPGL was defined as the recurrence at sites without chromaffin tissue [9]. All recorded laboratory indicators were the results of the patients before surgery. Measurements of plasma normetanephrine, plasma metanephrine, and 24 h urinary catecholamines were by mass spectrometry. Plasma metanephrine and plasma normetanephrine were measured after the patients maintained a supine position for at least 30 min [1]. Factors that affect catecholamine levels, such as caffeine, tricyclic antidepressants, phenoxybenzamine, sympathomimetics, and monoamine oxidase inhibitors, were discontinued at least 24 h before blood samples were obtained [1,10,11]. The tumor diameters were determined on the basis of the pathological specimens. Most PHEOs were resected with minimally invasive adrenalectomy; however, open resection was performed for large tumors (>6 cm). Most PGLs were resected with open surgery; however, laparoscopic resection was performed for small tumors in surgically favorable locations [1]. All patients with hormonally functional PPGLs underwent preoperative blockade, and the α-adrenergic receptor blockers were the first choice. The β-adrenergic receptor blockers were indicated only after administration of α-adrenergic receptor blockers [1]. If patients with negative catecholamines had positive functional imaging findings, they also underwent preoperative preparation as described above. If these patients were with negative functional imaging results, a decision about whether to use preoperative preparation was made by multidisciplinary teamwork [12].

The study was approved by the ethics committee of Peking Union Medical College Hospital and was conducted in accordance with the Declaration of Helsinki. The in-formed consent requirement was waived because all data were anonymized.

Statistical methods

Histograms and normal quantile–quantile plots were used to assess normality. Continuous data were reported as the mean ± standard or median (25th, 75th percentiles), and they were compared between the groups by Student’s *t*-test or the rank-sum test. Categorical variables are presented as numbers (percentages) and were compared using the Pearson’s chi-square test or Fisher’s exact test as appropriate. Parameters with *p* < 0.1 in the univariate logistic regression analysis were included in the multivariate logistic regression analysis. Two-sided *p* value < 0.05 was considered as statistically significant. Statistical analyses were performed using SPSS statistical software, version 25.0 (IBM Corp., Armonk, NY, USA). GraphPad Prism 8.0 (GraphPad, San Diego, CA, USA) was used to perform receiver operating characteristic (ROC) curve analysis.

## 3. Results

Among the 214 study patients, 69 patients had negative catecholamine levels. The patients’ clinical characteristics are summarized in Table 1. The mean age of the entire study population was 46.01 ± 12.95 years. Hypertension and diabetes accounted for 63.6% and 26.6% of the patients, respectively. Incidentaloma occurred in 79 patients (36.9%) in the entire cohort. Tumor location was extra-adrenal in 93 patients (43.5%) and adrenal in 121 (56.5%). Among extra-adrenal tumors, in 25 patients (26.9%) they were located in head and neck, and in 68 (73.1%) they were located in the thorax or abdomen. Open resection and minimally invasive adrenalectomy were performed in 142 patients (66.4%) and 72 patients (33.6%), respectively.

The clinical characteristics of patients with negative and positive catecholamines are summarized in Table 2. Age, sex, and BMI were not significantly different between the two groups. Fewer patients in the negative-catecholamine group had hypertension and diabetes compared with the positive-catecholamine group (43.5% vs. 73.1%, *p* < 0.001 and 10.1% vs. 34.5%, *p* < 0.001, respectively). Concentrations of total cholesterol, triglycerides, and low-density lipoprotein cholesterol did not significantly differ between the groups. In the negative-catecholamine group, median concentrations of plasma metanephrine, plasma normetanephrine, 24 h urine epinephrine, and 24 h urine norepinephrine were 0.1 nmol/L, 0.27 nmol/L, 3.37 μg/24 h, and 28.39 μg/24 h, respectively. Corresponding concentrations in the positive-catecholamine group were 0.39 nmol/L, 3.77 nmol/L, 5.68 μg/24 h, and 126.00 μg/24 h, respectively. Extra-adrenal PPGLs were more frequent in the negative-catecholamine group compared with the positive-catecholamine group (65.2% vs. 33.1%, *p* < 0.001). In patients with head and neck PPGLs, 2 patients (4.2%) were in the positive-catecholamine group and 23 patients (51.1%) were in the negative-catecholamine group (*p* < 0.001). In patients with thoracic or abdominal PPGLs, 46 patients (95.8%) were in the positive-catecholamine group and 22 patients (48.9%) were in the negative-catecholamine group (*p* < 0.001). Tumor diameter in the negative-catecholamine group was significantly smaller than that in the positive-catecholamine group (4.0 (3.0, 6.0) vs. 5.5 (4.5, 7.0) cm, *p* < 0.001).

**Figure 1:**
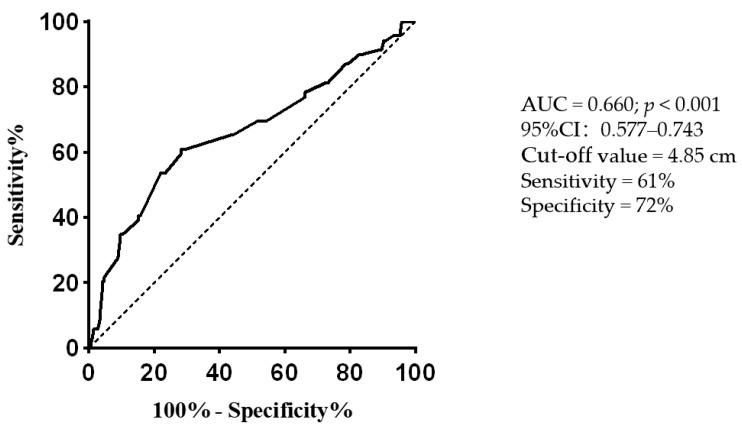
Receiver operating characteristic curve evaluating the diagnostic potential of tumor diameter for predicting PPGLs with negative catecholamines. AUC: area under the curve; PPGLs: pheochromocytomas and paragangliomas.

In the univariate logistic regression analysis, extra-adrenal PPGLs were positively associated with negative catecholamines (odds ratio (OR): 3.789, 95% confidence interval (95% CI): 2.071–6.933; *p* < 0.001). Diabetes, hypertension, and tumor diameter were negatively associated with negative catecholamines (OR: 0.215, 95% CI: 0.091–0.504, *p* < 0.001; OR: 0.283, 95% CI: 0.155–0.516, *p* < 0.001; and OR: 0.77, 95% CI: 0.662–0.895, *p* = 0.001, respectively). These results are summarized in Table 3. According to the results of the univariate logistic regression analysis, diabetes, hypertension, total cholesterol, extra-adrenal PPGL, and tumor diameter were included in the multivariate logistic regression analysis. The results showed that extra-adrenal PPGL (OR, 2.724; 95% CI: 1.382–5.372; *p* = 0.004) was independently positively associated with negative catecholamines; hypertension (OR, 0.305, 95% CI: 0.155–0.600, *p* = 0.001) and tumor diameter (OR, 0.826, 95% CI: 0.707–0.966, *p* = 0.016) were independently negatively associated with negative catecholamines. We used ROC curves to determine the diagnostic potential of tumor diameter for PPGLs with negative catecholamines. The area under the curve was 0.660 (95% CI: 0.577–0.743; *p* < 0.001), and the cutoff value was 4.85 cm (Figure 1).

In this study, 180 patients were followed up for a mean of 20.54 ± 11.83 months, including 61 in the negative-catecholamine group and 119 in the positive-catecholamine group. Among them, five patients developed disease recurrence, namely three in the negative-catecholamine group and two in the positive-catecholamine group. There was no significant difference in tumor recurrence rates between the groups (*p* = 0.44). Three patients were diagnosed with metastases during the follow-up, and all were in the positive-catecholamine group. One patient died because of hypertension in the positive-catecholamine group.

## 4. Discussion

In our study, a positive association was found between extra-adrenal PPGLs and negative catecholamines, and there was no significant difference in early tumor recurrence rates between the two groups. Additionally, comorbidities were less frequent and tumor diameter was smaller in the negative-catecholamine group. This study provided useful information for clinicians to understand the PPGL patients with negative catecholamines, which was very helpful for diagnosis and follow-up of patients with PPGLs.

A previous study of 42 patients presenting with adrenal incidentaloma revealed 14 cases of PHEO, with 3 (21%) of these exhibiting borderline urine or serum metanephrine concentrations [13]. Another study from Italy revealed that 14% of the patients with PHEOs had negative urine catecholamines [14]. In Kawashima et al.’s cohort [15], the prevalence of patients with PPGLs and negative urine catecholamine results was 6.2%. In Heavner et al.’s study [6], 9% of patients with PHEOs had negative markers preoperatively. On the basis of these findings, the exact proportion of negative catecholamines in patients with PPGLs is not yet clear. Two factors may explain the wide range of reported rates: study population and definition of negative catecholamine concentrations. The evaluated population in our study comprised patients with PPGLs; however, some previous studies evaluated patients with PHEOs only [6,14]. Furthermore, the definition of negative catecholamines varied in previous studies in accordance with testing conditions at the different medical facilities where the studies were conducted. In our study, negative catecholamine was defined as plasma metanephrine, plasma norepinephrine, and urinary catecholamine concentrations not exceeding their respective upper reference limits. In Kawashima et al.’s study [15], negative catecholamine was defined as when the levels of urinary metanephrine and normetanephrine did not exceed their upper reference limits. Large-scale, well-defined, and well-targeted studies are needed to address this issue; however, because of the rarity of PPGLs, performing these studies will be a great challenge.

In the present study, extra-adrenal PPGLs were significantly associated with negative catecholamines, and this result was similar with Kawashima et al.’s study [15]. An association between negative catecholamines and extra-adrenal PPGLs is implied by the high proportion of extra-adrenal PPGLs in patients in the negative-catecholamine group. In PPGLs with the *SDHB* mutation, tyrosine hydroxylase is sometimes absent, resulting in PPGL with biochemical silence [16]. Moreover, biochemically silent PPGLs have been associated with *SDHD* mutations in a previous study [17]. A recent paper describing the natural history and management of familial PGL syndrome type 1 also reported that negative biochemical results occurred in the patients with *SDHD* mutations [18]. In addition, according to Neumann et al.’s study, patients with *SDHB/SDHD* mutations were significantly more likely to develop extra-adrenal PPGL than those without [19]. According to the results of above studies, the tumor locations and catecholamine secretion may be associated with the type of gene mutation.

In a recent systematic review reporting patients with PPGLs treated with Sunitinib, almost half of the patients with malignant PPGLs did not have excess catecholamine secretion, while the remaining patients were with elevated catecholamines [20]. This phenomenon suggests that it is very interesting to explore the relationship between catecholamine secretion and metastatic progression of PPGLs. In Kawashima et al.’s study [15], PPGLs with negative catecholamines were associated with metastatic disease. In contrast to Kawashima et al.’s results [15], Heavner et al. [6] reported there were no metastatic PPGLs in patients with negative catecholamines, whereas there were nine metastatic cases in patients with positive catecholamines. Another study also reported that catecholamine concentrations were higher in patients with metastatic PPGLs than non-metastatic PPGLs [21]. In our study, after the short-term follow-up, only three patients were diagnosed with metastatic PPGLs, and all were in the catecholamine-positive group. Compared with previous reports [15,22,23], the proportion of metastatic PPGLs was lower in this study. The possible reasons for this difference are as follows: First, we excluded patients who presented with recurrence or metastasis of PPGL after treatment in other hospitals before analysis. Second, as metastatic PPGLs often become evident several years after initial diagnosis, the lower metastatic prevalence in this study may be due in part to the short-term follow-up. Nonetheless, the proportion of tumor recurrence between the two groups was not significantly different, suggesting that it is essential to closely monitor patients in the catecholamine-negative group for tumor recurrence, just as patients with positive catecholamines.

Several previous studies have reported a positive correlation between tumor size and catecholamine concentrations [24,25,26]. In this study, we also found that tumor diameter in the patients in the negative-catecholamine group was smaller than that in the positive-catecholamine group. Although tumor diameter was smaller in patients with negative catecholamines, existing literature has indicated that caution should be exercised regarding complications when resecting these tumors. In one case report, a hypertensive episode occurred during resection of an incidentally discovered adrenal lesion in a patient without elevated metanephrine concentration, and PHEO was later diagnosed [7]. Despite successful treatment, this case illustrates that complications may still occur during surgical resection of tumors with negative catecholamines.

In our study, hypertension and diabetes were less frequent in the negative catecholamine group than the positive catecholamine group, which was expected owing to the effect of catecholamines on blood pressure and glucose metabolism [27,28,29]. Catecholamines also affect body weight during hypermetabolic and proinflammatory states [30]. As a result of a comparison between patients with negative catecholamines and patients with catecholamine-positive PPGLs, Heavner et al. [6] reported that BMI was higher in patients with negative catecholamines; however, Kawashima et al. [15] did not find a difference in BMI between their negative- and positive-catecholamine groups, and the results in this study were consistent with Kawashima et al.’s. The difference between the BMI in the above studies may be due to the different prevalence of obesity between Asians and Americans.

Anatomical documentation of the tumor is necessary to diagnose PPGLs, and hormonal tests for catecholamines are helpful in the diagnosis of them [11]. Current Endocrine Society Guidelines [1] suggests annual biochemical surveillance for PPGL patients. According to Puliani et al.’s suggestions [18], in PPGL patients with negative biochemical results and *SDHD* mutations, periodic follow-up should include an annual biochemical and ultrasonographic screening and biannual neck-mediastinum magnetic resonance examination. Based on our experience, for catecholamine-negative patients, we also recommend annual biochemical testing and ultrasonographic screening, as well as biannual magnetic resonance imaging to assess recurrence and metastasis.

This study has several limitations. First, bias was inevitable because of the retrospective and single-center study design. Second, plasma metanephrine and plasma normetanephrine concentrations were not measured in all patients. However, not all hospitals have the ability to measure plasma-free catecholamines, while measurement of urine catecholamines is common and feasible. Third, owing to the lack of genetic screening, we could not confirm a relationship between genotype and catecholamines. Fourth, there was no reliable method for dopamine-producing tumors. The plasma methoxytyramine measurement was not available in our medical institution; although urinary dopamine was collected, the majority of it is synthesized in the renal tubules from circulating Dopa. Therefore, urinary dopamine is not a reliable indicator of dopamine-producing tumors. As tumors that produce dopamine predominantly or exclusively are rare [31,32,33], the results in our study can still be used for the assessments of most PPGLs.

## 5. Conclusions

The existence of catecholamine-negative PPGLs has been established, and they are not uncommon. Negative first-line catecholamine testing does not necessarily rule out a diagnosis of PPGLs. PPGL patients with negative catecholamines had an increased likelihood of having extra-adrenal lesions and a lower likelihood of having comorbidities. In addition, patients with preoperative negative catecholamines should be closely monitored for tumor recurrence.

## Figures and Tables

**Table 1:** Clinical characteristics of patients with pheochromocytoma and paragangliomas.

Variable	All (*n* = 214)
Age, years (*n* = 214)	46.01 ± 12.95
Female, % (*n* = 214)	112(52.3)
Diabetes, % (*n* = 214)	57(26.6)
Hypertension, % (*n* = 214)	136(63.6)
BMI, kg/m^2^ (*n* = 214)	24.37 ± 3.27
Metabolic parameters	
Glucose, mmol/L (*n* = 214)	5.4(4.7, 6.53)
Total cholesterol, mmol/L (*n* = 211)	4.49(4.02, 5.23)
Triglyceride, mmol/L (*n* = 211)	1.25(0.85, 1.79)
LDL-c, mmol/L (*n* = 210)	2.76 ± 0.76
Plasma metanephrine, nmol/L (*n* = 118)	0.18(0.1, 2.59)
Plasma normetanephrine, nmol/L (*n* = 118)	2.50(0.79, 5.98)
24hU-E, μg/24 h (*n* = 214)	4.22(2.81, 17.51)
24hU-NE, μg/24 h (*n* = 214)	56.88(31.81,188.01)
24hU-DA, μg/24 h (*n* = 214)	232.15(186.74, 296.01)
Tumor characteristics (*n* = 214)	
Adrenal PPGL, %	121(56.5)
Extra-adrenal PPGL, %	93(43.5)
Tumor diameter (cm)	5.0(4.0, 6.53)

PPGL, pheochromocytoma and paraganglioma; BMI, body mass index; 24hU-E: 24 h urine epinephrine; 24hU-NE: 24 h urine norepinephrine; 24hU-DA: 24 h urine dopamine; LDL-c, low-density lipoprotein cholesterol. Reference range: plasma metanephrine: <0.5nmol/L; plasma normetanephrine: <0.9nmol/L; 24hU-E: 1.74–6.42 μg/24 h; 24hU-NE: 16.69–40.65 μg/24 h; 24hU-DA: 120.93–330.59 μg/24 h.

**Table 2:** Clinical characteristics of the patients in the positive- and negative-catecholamine groups.

Variable	Negative-Catecholamine Group (*n* = 69)	Positive-Catecholamine Group (*n* = 145)	*p* Value
Age, years (*n* = 214)	47.91 ± 12.53	45.10 ± 13.09	0.138
Female, % (*n* = 214)	41(59.4)	71(49.0)	0.152
Diabetes, % (*n* = 214)	7(10.1)	50(34.5)	<0.001
Hypertension, % (*n* = 214)	30(43.5)	106(73.1)	<0.001
BMI, kg/m^2^ (*n* = 214)	24.63 ± 2.98	24.26 ± 3.40	0.439
Metabolic parameters			
Glucose, mmol/L (*n* = 214)	4.9(4.6, 5.65)	5.6(4.9, 6.75)	0.001
Total cholesterol, mmol/L (*n* = 211)	4.38(3.96, 4.92)	4.55(4.03, 5.34)	0.103
Triglyceride, mmol/L (*n* = 211)	1.36(0.90, 1.86)	1.23(0.76, 1.76)	0.36
LDL-c, mmol/L (*n* = 210)	2.63 ± 0.74	2.81 ± 0.77	0.106
Tumor characteristics (*n* = 214)			
Adrenal PPGL, %	24(34.8)	97(66.9)	<0.001
Extra-adrenal PPGL, %	45(65.2)	48(33.1)	<0.001
Tumor diameter (cm)	4.0(3.0, 6.0)	5.5(4.5, 7.0)	<0.001

PPGL, pheochromocytoma and paraganglioma; BMI, body mass index; LDL-c, low-density lipoprotein cholesterol.

**Table 3:** Results of the univariate logistic regression.

Variable	*p*	OR	95% CI
Age	0.139	1.017	0.994–1.040
Female	0.153	1.526	0.854–2.727
Diabetes	<0.001	0.215	0.091–0.504
Hypertension	<0.001	0.283	0.155–0.516
BMI	0.437	1.035	0.948–1.130
Total cholesterol	0.09	0.759	0.551–1.044
Triglyceride	0.747	0.963	0.768–1.209
LDL-c	0.108	0.725	0.489–1.073
Extra-adrenal PPGL	<0.001	3.789	2.071–6.933
Tumor diameter	0.001	0.77	0.662–0.895

PPGL, pheochromocytoma and paraganglioma; BMI, body mass index; LDL-c, low-density lipoprotein cholesterol; OR, odds ratio; 95% CI, 95% confidence interval.

## Data Availability

The data that support the findings of this study are available from the corresponding author upon reasonable request.
